# Supplementary material for: Extraction of alumina from alumina rich coal gangue by a hydro-chemical process
Source: R Soc Open Sci. 2020 Apr 29;7(4):192132. doi: 10.1098/rsos.192132 (PMC7211850; doi:10.1098/rsos.192132)
Supplement: Supplementary material [file rsos192132supp1.zip › electronic supplementary material/ESM file titles and captions .docx]

ESM file titles and captions

**1. Original source data**

**Title 1:** XRD data of the ARCG

**Legend/Caption 1:** data for Fig.1

**Title 2:** data of alkali concentration versus extraction rate of Al_2_O_3_

**Legend/Caption 2:** data for Fig.3

**Title 3:** XRD data of ARCG residue obtained at different alkali concentration

**Legend/Caption 4:** data for Fig.4

**Title 4** data of alkali to gangue ratios versus extraction rate of Al_2_O_3_

**Legend/Caption 5:** data for Fig.5

**Title 5:** XRD data of ARCG residue obtained at different alkali to gangue ratio

**Legend/Caption 6:** data for Fig.6

**Title 6:** data of reaction temperature versus extraction rate of Al_2_O_3_.

**Legend/Caption 7:** data for Fig.7

**Title 7:** XRD data of ARCG residue obtained at different temperatures.

**Legend/Caption 8:** data for Fig.8

**Title 8:** XRD data of ARCG residue obtained at different reaction time

**Legend/Caption 9:** data for Fig.9

**Title 9:** XRD data of ARCG residue obtained under optimum condition

**Legend/Caption2:** data for Fig.10

**Title 10:** data of 1−(1−x )^1/3^ versus time

**Legend/Caption 12:** data for Fig.12

**Title 11:** Arrhenius data for alumina extraction during 200 ℃ to 280 ℃.

**Legend/Caption 13:** data for Fig.13

**2. Electronic file of each table**

**Title 1:** Table 1

**Legend/Caption 1:** data for Table 1

**Title 2:** Table 2

**Legend/Caption 1:** data for Table2

**3. Electronic file of each figure**

**Title 1:** Fig.1

**Legend/Caption 1:** electronic file of Fig.1

**Title 2:** Fig.2

**Legend/Caption 2:** electronic file of Fig.2

**Title 3:** Fig.3

**Legend/Caption 4:** electronic file of Fig.3

**Title 4** : Fig.4

**Legend/Caption 5:** electronic file of Fig.4

**Title 5:** Fig.5

**Legend/Caption 6:** electronic file of Fig.5

**Title 6:** Fig.6

**Legend/Caption 7:** electronic file of Fig.6

**Title 7:** Fig.7

**Legend/Caption 8:** electronic file of Fig.7

**Title 8:** Fig.8

**Legend/Caption 9:** electronic file of Fig.8

**Title 9:** Fig.9

**Legend/Caption2:** electronic file of Fig.9

**Title 10:** Fig.10

**Legend/Caption 12:** electronic file of Fig.10

**Title 11:** Fig.11

**Legend/Caption 13:** electronic file of Fig.11

**Title 12:** Fig.12

**Legend/Caption 12:** electronic file of Fig.12

**Title 13:** Fig.13

**Legend/Caption 13:** electronic file of Fig.13

**4. Manuscript**

**Title 1:** main document

**Legend/Caption 1:** manuscript
